# Deadly Marburg virus outbreak received sustained attention: What can we learn from the existing studies?

**DOI:** 10.1097/JS9.0000000000000443

**Published:** 2023-05-18

**Authors:** Kunming Cheng, Qiang Guo, Shuqin Gu, Haiyang Wu, Cheng Li

**Affiliations:** a Department of Intensive Care Unit, The Second Affiliated Hospital of Zhengzhou University, Zhengzhou, Henan; b Department of Orthopedics, Baodi Clinical College of Tianjin Medical University; c Department of Graduate School, Tianjin Medical University, Tianjin; d Department of Orthopaedic Surgery, Beijing Jishuitan Hospital, Fourth Clinical College of Peking University, Beijing, People’s Republic of China; e Duke Molecular Physiology Institute, Duke University School of Medicine; f Duke Human Vaccine Institute, Duke University Medical Center, Durham, North Carolina, USA; g Center for Musculoskeletal Surgery (CMSC), Charité-Universitätsmedizin Berlin, corporate member of Freie Universität Berlin, Humboldt University of Berlin, and Berlin Institute of Health, Berlin, Germany

*Dear Editor,*

Marburg virus disease (MVD), formerly known as Marburg hemorrhagic fever, is a serious and frequently fatal illness in humans caused by the Marburg virus (MARV), which belongs to the *Filoviridae* family along with another more notorious Ebola virus. MARV has the characteristics of high infectivity and a fatality rate of up to 80–90%. As early as 2015, World Health Organization (WHO) listed it as a key infectious disease that might cause a pandemic and required high attention. And in 2018, WHO further placed it on the list of pathogens with epidemic and the Public Health Emergency of International Concern (PHEIC) potential. Meanwhile, the 2014–2016 outbreak of Ebola virus disease in West Africa made MARV also a global concern together, which promoted the development of prevention and control work, and corresponding scientific research and diagnostic products. Recently, WHO successively announced the outbreak of MARV in Equatorial Guinea and the United Republic of Tanzania. This highly lethal virus once again became a hot topic of the whole society^1,2^. However, to the best of our knowledge, there is still lacking a detailed report to summarize the current state and research hotspots of the field. In view of this, the current study was designed to perform a systematic bibliometric analysis of the countries, institutions, authors, keywords, and cited literature in this field for providing a reference for future research communication and cooperation.

As a consequence of the coronavirus disease 2019 (COVID-19) pandemic, the field of infectious diseases has been a considerable contributor to biomedical literature and one of the most exciting areas for bibliometric analysis^3^. In this study, the document retrieval was restricted to the terms like ‘Marburg virus*’, ‘Marburgvirus’, ‘Marburg haemorrhagic fever’, ‘Marburg hemorrhagic fever’ from the Web of Science Core Collection (WoSCC) on 10 April 2023. There are no restrictions for a retrieval time frame, and only ‘original research articles or review articles’ published in English were considered for data analysis. After initial screening and duplicate removal, an online analysis platform, CiteSpace 6.2.R2, and VOSviewer 1.16.16 software were used for bibliometric analysis.

A total of 880 qualified publications, including 759 articles and 121 reviews, were finally identified. The annual distribution of publication number and citation frequency is available in Figure 1A. It can be observed that the first study with the term MARV was published in 1968. As a matter of fact, it was first recognized after simultaneous outbreaks in Marburg and Frankfurt, Germany, in 1967, when workers at the research laboratory were infected by nonhuman primate animal products. From the point of view of literature outputs, MARV has not received much scientific attention before 2000, with no more than 10 studies each year. When the time to the 21st century, especially between 2000 and 2015, the world’s research in this field showed a rapid growth trend. In order to figure out the reasons for this tendency, we have marked the year, regions, infected cases, and cumulative deaths of the observed MARV outbreak around the world. It is possible to see that since the beginning of the 21st century, the outbreak frequency and severity levels have been much higher than before. This may be the possible reason why MARV acquires increasing attention. In addition, in order to compare with another *Filoviridae* family member of the Ebola virus, we also searched all related studies on the Ebola virus from WoSCC. As can be seen from Supplementary Figure 1 (Supplemental Digital Content 1, http://links.lww.com/JS9/A522), a total of 8174 documents on the Ebola virus was identified, and since 2015, the annual number of publications was more than 500. From this result, it is not difficult to see that MARV-related studies have not received sufficient attention over the past years. Therefore, our results strongly appeal to more extensive studies of MARV to better respond to this potential threat to public health in the future.

**Figure 1:**
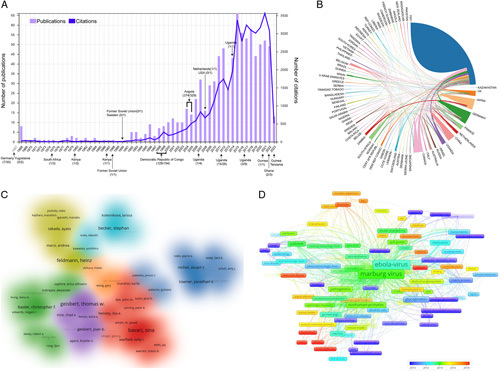
(A) The annual distribution of publication number and citation frequency; (B) a national cooperation network map; (C) cluster map of author co-authorship analysis; (D) keyword co-occurrence analysis.

In total, 79 countries/regions contributed studies in this area, with the United States leading the list with 581 documents (66%), followed by Germany with 160 (18%) and Canada with 73 (8%) (Supplementary Figure 2, Supplemental Digital Content 2, http://links.lww.com/JS9/A523). A national cooperation network map showed that the United States collaborated most closely with Canada, Germany, and Uganda, while Japan also collaborated closely with Zambia (Fig. 1B). Nevertheless, other countries, especially developing countries, lack such international cooperation. In order to help scholars looking for potential collaborators, we have further analyzed the active researchers and institutions in this field. As shown in Figure 1C, a total of seven research clusters (with the same color) were found. Among them, Feldmann H., the Chief Scientist of the BSL4 Laboratories at the Rocky Mountain Laboratories, contributed the largest number of documents, followed by Becker S. from Philipps University Marburg/German Center for Infection Research, Bavari S. and Geisbert T.W. from the United States Army Medical Research Institute of Infectious Diseases. While for author co-citation analysis (Supplementary Figure 3, Supplemental Digital Content 3, http://links.lww.com/JS9/A524), Geisbert T.W. has the highest number of citations, followed by Feldmann H., and Towner J.S. from Centers for Disease Control and Prevention. In terms of institution analysis, the National Institute of Allergy Infectious Diseases (NIAID), the University of Texas Medical Branch, and the Centers for Disease Control and Prevention have published the highest number of studies (Supplementary Figure 4, Supplemental Digital Content 4, http://links.lww.com/JS9/A525). In addition, we also summarized the top 10 funding agencies in this field in Supplementary Table 1 (Supplemental Digital Content 5, http://links.lww.com/JS9/A526). As expected, funding agencies from the United States funded the greatest number of studies. All in all, from the distribution of counties, authors, institutions, and funding agencies, the United States has made a tremendous contribution to this area, which cannot be separated from the support of sufficient funding and outstanding scientific researchers. Predictably, the above-mentioned counties, authors, and institutions may also be the major promoters of MARV research in the future.

In general, the number of citations of one individual article could reflect its academic importance and popularity in the scientific community. We have summarized the top 15 highly cited studies related to MARV in Supplementary Table 2 (Supplemental Digital Content 6, http://links.lww.com/JS9/A527). All of them were published between 1999 and 2015, with at least 259 citation times. After analyzing the main topics of all these studies, the following research directions, including preventive vaccines, antiviral treatments such as BCX4430 and Tetherin, MARV hosts such as Egyptian fruit bats and African bats, PCR rapid detection, the transmission, transcription and replication mechanism of MARV, have received relatively much attention. Moreover, to better understand the developmental process of MARV research, references with strong citation bursts were identified by CiteSpace. Supplementary Figure 5 (Supplemental Digital Content 7, http://links.lww.com/JS9/A528) displayed the top 25 references with the strongest citation bursts from 2000 to 2023. Among them, reference with citation burst first emerged in 2005 owing to one study published by Jones *et al.* ^4^, about the protective role of live attenuated recombinant vaccine based on attenuated recombinant vesicular stomatitis virus (rVSV) vectors in nonhuman primate models. Additionally, two studies with ongoing bursts until 2023 deserve more attention. Of them, one study by Mire *et al*.^5^ found a human monoclonal antibody called MR191-N could confer a survival benefit of up to 100% to Marburg virus-infected rhesus macaques when treatment was initiated up to 5 days postinoculation. Overall, the development of a safe and effective vaccine as well as an antiviral therapy program has been of high priority in MARV research. From the present perspective, adenoviral or rVSV-vectored vaccines are the most promising approach. While BCX4430 and MR191-N, as promising potential therapeutic agents, are currently undergoing clinical development^6,7^.

Apart from references, keyword analysis is another commonly used method to analyze the current hotspots and future frontiers of a specific research area. Generally speaking, the occurrence frequency is often used as the criteria to quantify keyword popularity. As shown in Figure 1D, keywords with relatively high word occurrence frequency were ‘Marburg virus’, ‘Ebola-virus’, ‘filovirus’, ‘hemorrhagic-fever’, ‘infection’, ‘nonhuman-primates’, ‘glycoprotein’, ‘outbreak’, ‘replication’, ‘pathogenesis’, ‘vaccine’, ‘antibodies’, ‘matrix protein’. This result is in line with the analysis from highly cited studies. Moreover, in this overlay map of Figure 1D, different keywords were marked with different colors according to the corresponding average appearing year (AAY). Therefore, according to the color gradient in the lower right corner, these keywords marked with red color could reflect the future research hotspots to a certain extent. Thus, keywords with a relatively latest AAY, such as ‘inhibitors’, ‘double-blind’, ‘safety’, ‘efficacy’, ‘immunogenicity’, ‘fruit bats’, and ‘neutralizing antibodies’ have great potential to become research focuses in the near future.

In conclusion, to our knowledge, this is the first study to conduct a comprehensive analysis of global research developments and hotspots in the field of MARV. Our results showed that although the number of publications related to MARV has increased significantly since the early 2000s, it still has not received as much focus as the Ebola virus. The current research topics mainly included preventive vaccines, antiviral treatments, host species, rapid detection methods, and understanding the transmission, transcription, and replication mechanisms of the virus. And the future research hotspots in MARV may involve the development of inhibitors, evaluation of safety and efficacy in clinical trials, understanding the immunogenicity of vaccines, studying fruit bats as potential reservoirs, and investigating neutralizing antibodies. In the context of many infectious diseases such as COVID-19, monkeypox, and cholera still ravaging the world, the frequent outbreaks of MARV in several countries could ring an alarm bell. As the old proverb states, take precautions beforehand, and provide against the future. Whether or not MARV will spread widely now or in the future, sustained research efforts and cooperation should be encouraged around the world.

## Ethical approval

This study does not include any individual-level data and thus does not require any ethical approval.

## Sources of funding

This study is supported by China Postdoctoral Science Foundation (2022M720385) and Beijing JST Research Funding (YGQ-202313).

## Author contribution

K.C.: conceptualization, methodology, data curation, formal analysis, resources, investigation, and writing – original draft; Q.G.: conceptualization, methodology, data curation, formal analysis, resources, and investigation; S.G: conceptualization, methodology, data curation, formal analysis, resources, and investigation; H.W.: conceptualization, methodology, data curation, formal analysis, resources, and investigation; C.L.: methodology, data curation, formal analysis, resources, investigation, and writing – review and editing.

## Conflicts of interest disclosure

The authors declare no conflicts of interest.

## Research registration unique identifying number (UIN)

1. Name of the registry: not applicable.
2. Unique identifying number or registration ID: not applicable.
3. Hyperlink to your specific registration (must be publicly accessible and will be checked): not applicable.

## Guarantor

Haiyang Wu and Cheng Li.

## Data availability statement

The data underlying this article will be shared by the corresponding author upon reasonable request.

## Supplementary Material
