# Supplementary material for: Deadly Marburg virus outbreak received sustained attention: What can we learn from the existing studies?
Source: Int J Surg. 2023 May 18;109(8):2539–41. doi: 10.1097/JS9.0000000000000443 (PMC10442097; doi:10.1097/JS9.0000000000000443)
Supplement: Supplementary file 1 [file js9-109-2539-s001.docx]

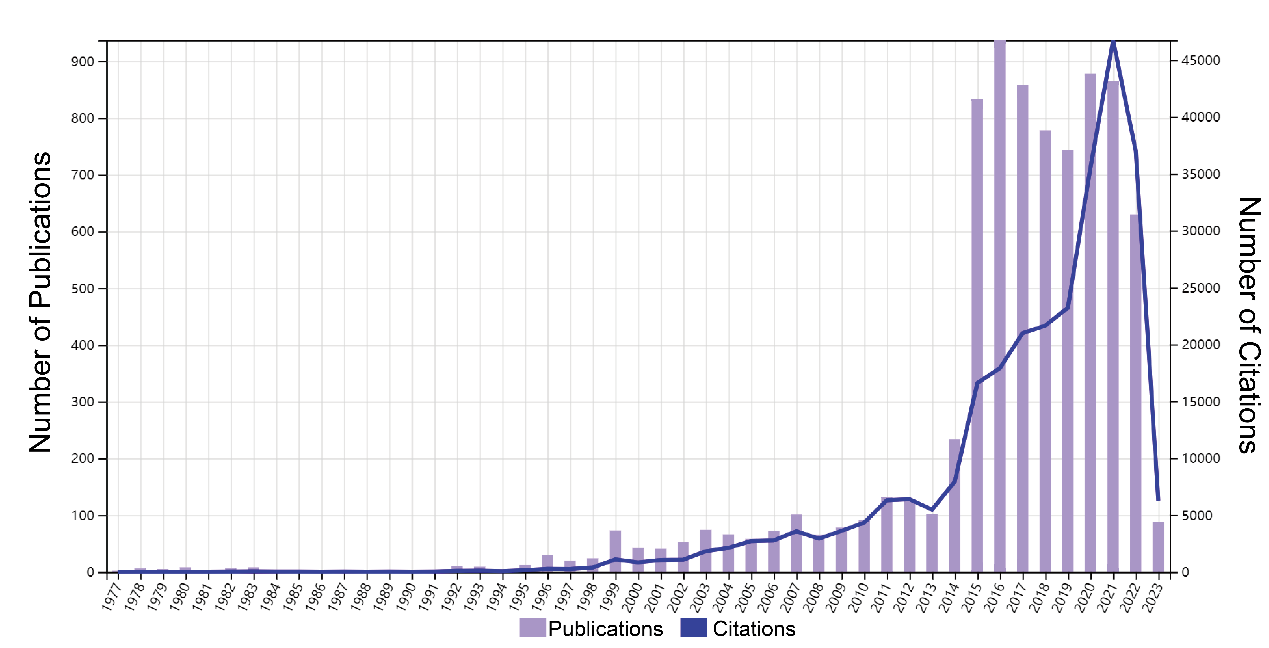


Supplementary Figure 1: The annual number of publications and citations related to Ebola virus from 1977 to 2023.
