# Supplementary figures and images for: Deadly Marburg virus outbreak received sustained attention: What can we learn from the existing studies?
Source: Int J Surg. 2023 May 18;109(8):2539–41. doi: 10.1097/JS9.0000000000000443 (PMC10442097; doi:10.1097/JS9.0000000000000443)

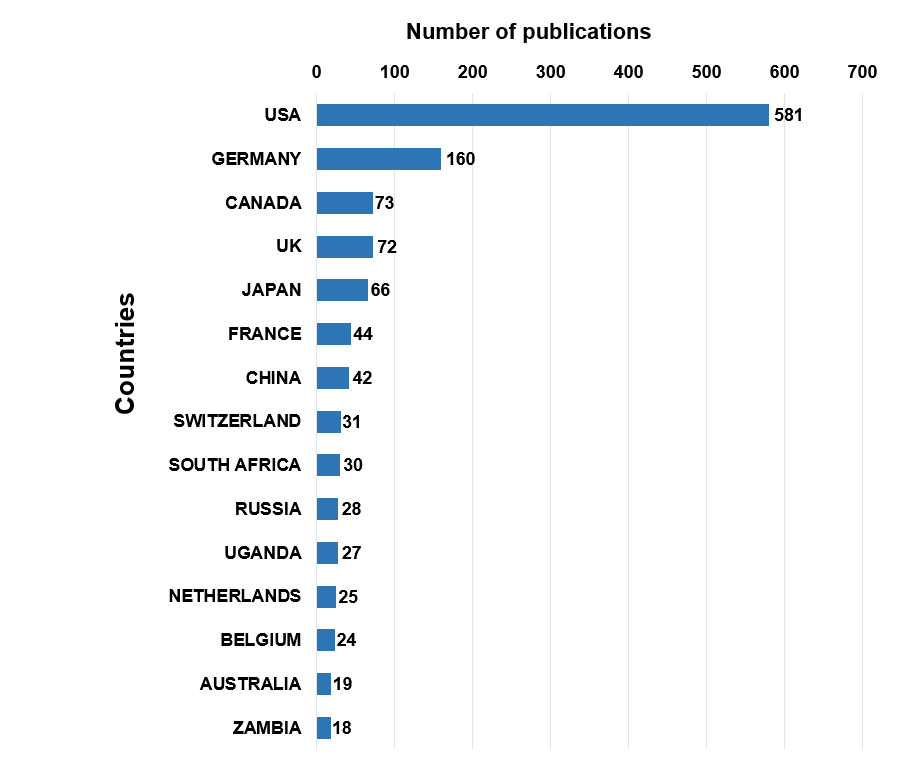


Supplementary Figure 2: The annual publication trend among the top 15 countries

Supplement: Supplementary file 2 [file js9-109-2539-s002.docx]

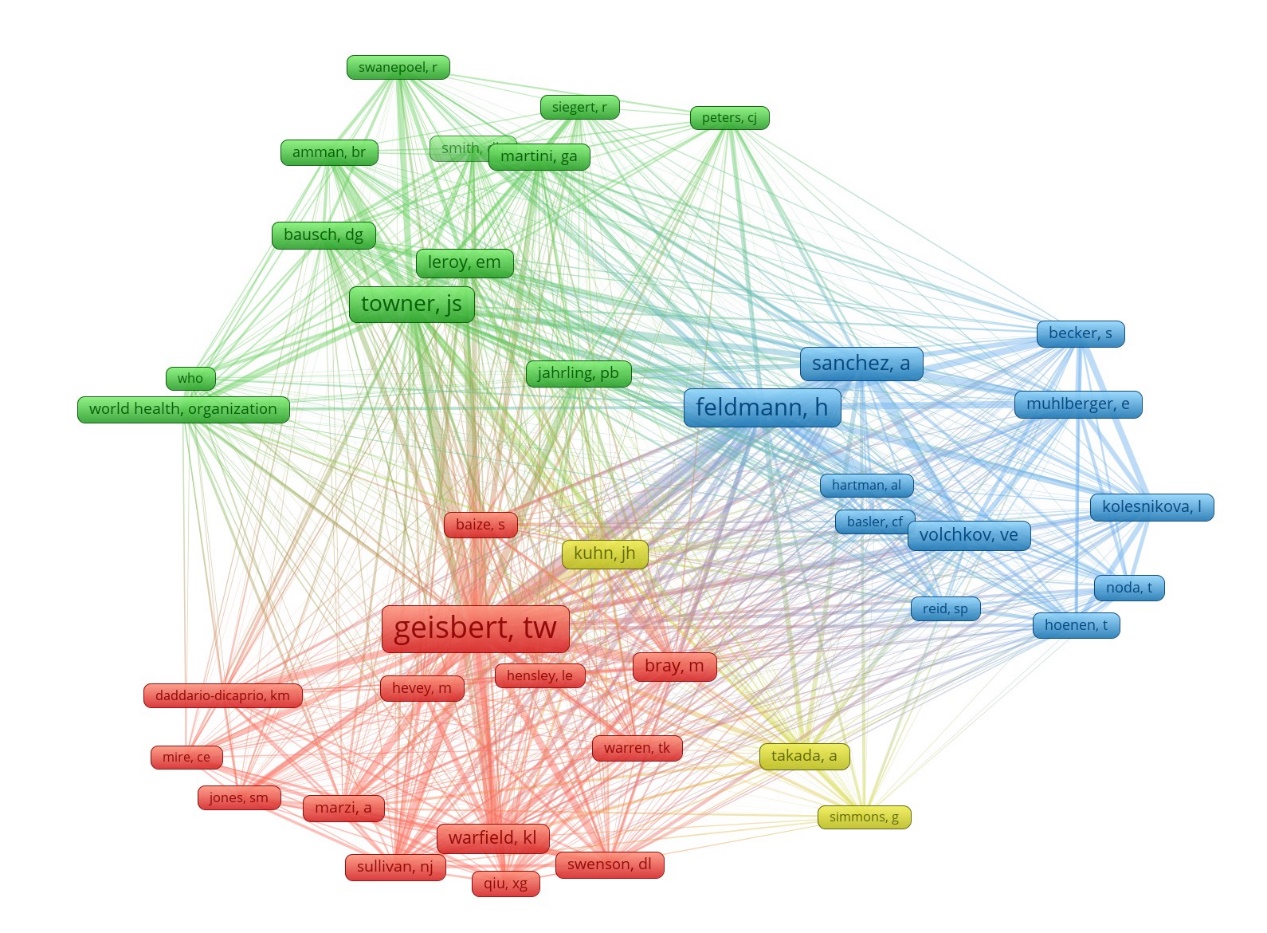


Supplementary Figure 3: Author co-citation analysis

Supplement: Supplementary file 3 [file js9-109-2539-s003.docx]

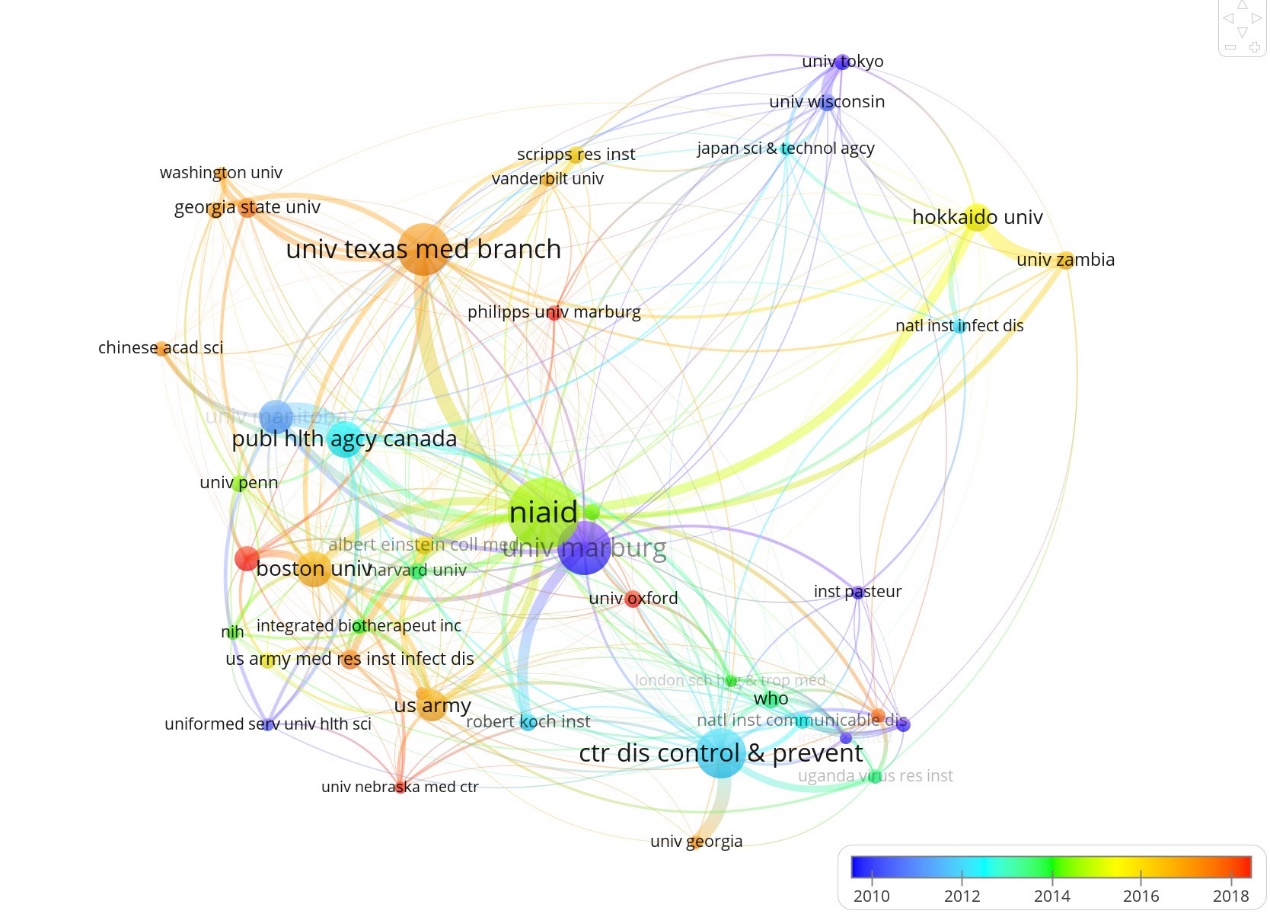


Supplementary Figure 4: Overlay visualization map of institution co-authorship analysis

Supplement: Supplementary file 4 [file js9-109-2539-s004.docx]

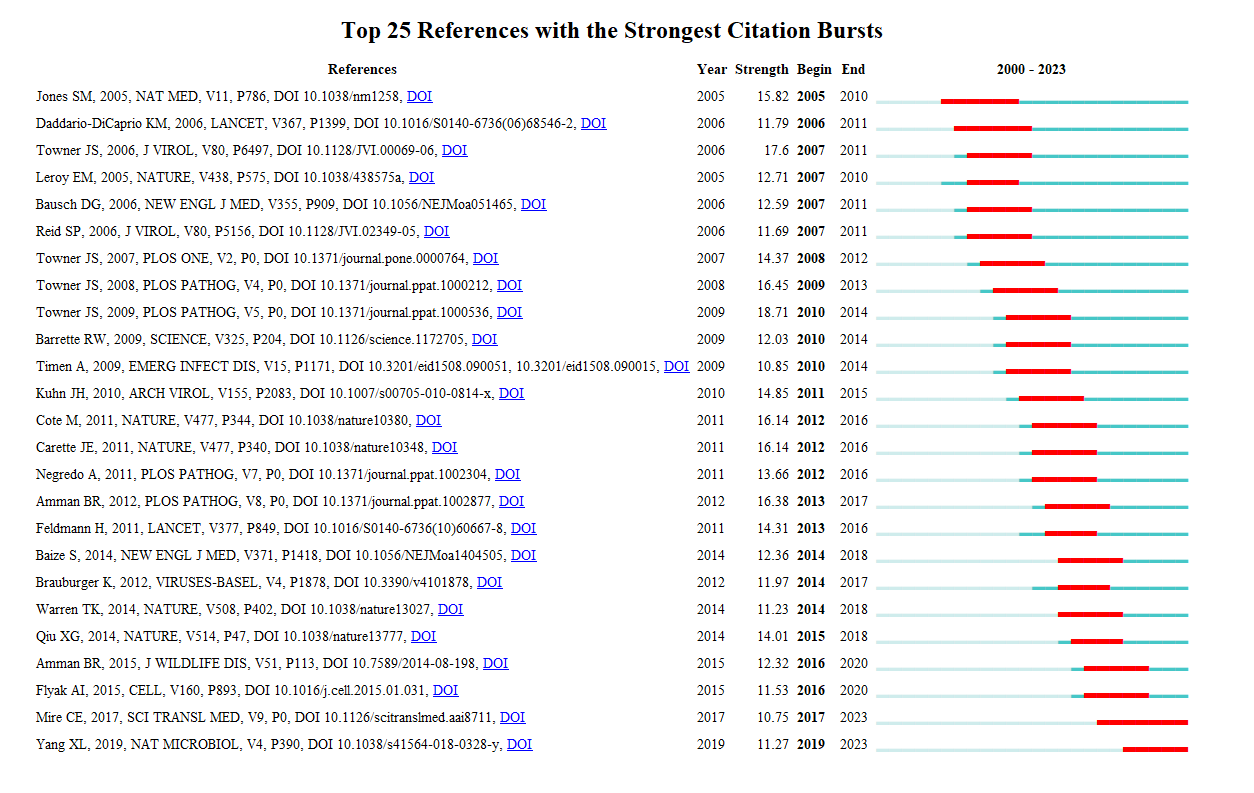


Supplementary Figure 5: Top 25 references with the strongest citation bursts

Supplement: Supplementary file 7 [file js9-109-2539-s007.docx]
