# Supplementary material for: Deadly Marburg virus outbreak received sustained attention: What can we learn from the existing studies?
Source: Int J Surg. 2023 May 18;109(8):2539–41. doi: 10.1097/JS9.0000000000000443 (PMC10442097; doi:10.1097/JS9.0000000000000443)
Supplement: Supplementary file 5 [file js9-109-2539-s005.docx]

Supplementary Table 1: The top 10 funding agencies in this field

| Funding agencies | N |
| --- | --- |
| United States Department of Health Human Services | 279 |
| National Institutes of Health | 269 |
| NIH National Institute of Allergy Infectious Diseases | 126 |
| United States Department of Defense | 96 |
| Defense Threat Reduction Agency | 86 |
| Ministry Of Education Culture Sports Science and Technology Japan | 40 |
| German Research Foundation | 39 |
| Japan Society for the Promotion of Science | 37 |
| Grants In Aid for Scientific Research Kakenhi | 28 |
| European Commission | 20 |
