# Supplementary material for: Deadly Marburg virus outbreak received sustained attention: What can we learn from the existing studies?
Source: Int J Surg. 2023 May 18;109(8):2539–41. doi: 10.1097/JS9.0000000000000443 (PMC10442097; doi:10.1097/JS9.0000000000000443)
Supplement: Supplementary file 6 [file js9-109-2539-s006.docx]

Supplementary Table 2: Top 15 highly cited studies in Marburg viruses field

| Ranking | Title | Total citations | AC per year | Journal | First Author | Published  year |
| --- | --- | --- | --- | --- | --- | --- |
| 1 | Ebola virus entry requires the cholesterol transporter Niemann-Pick C1 | 858 | 66 | NATURE | Carette, Jan E | 2011 |
| 2 | Live attenuated recombinant vaccine protects nonhuman primates against Ebola and Marburg viruses | 485 | 25.53 | NATURE MEDICINE | Jones, SM | 2005 |
| 3 | Rapid detection and quantification of RNA of Ebola and Marburg viruses, Lassa virus, Crimean-Congo hemorrhagic fever virus, Rift Valley fever virus, Dengue virus, and Yellow fever virus by real-time reverse transcription-PCR | 452 | 20.55 | JOURNAL OF CLINICAL MICROBIOLOGY | Drosten, C | 2002 |
| 4 | Isolation of Genetically Diverse Marburg Viruses from Egyptian Fruit Bats | 397 | 26.47 | PLOS PATHOGENS | Towner, Jonathan S | 2009 |
| 5 | Protection against filovirus diseases by a novel broad-spectrum nucleoside analogue BCX4430 | 393 | 39.3 | NATURE | Warren, Travis K | 2014 |
| 6 | Lipid raft microdomains: A gateway for compartmentalized trafficking of Ebola and Marburg viruses | 363 | 16.5 | JOURNAL OF EXPERIMENTAL MEDICINE | Bavari, S | 2002 |
| 7 | Comparison of the transcription and replication strategies of Marburg virus and Ebola virus by using artificial replication systems | 360 | 14.4 | JOURNAL OF VIROLOGY | Muhlberger, E | 1999 |
| 8 | A PPxY motif within the VP40 protein of Ebola virus interacts physically and functionally with a ubiquitin ligase: Implications for filovirus budding | 359 | 14.96 | PROCEEDINGS OF THE NATIONAL ACADEMY OF SCIENCES OF THE UNITED STATES OF AMERICA | Harty, RN | 2000 |
| 9 | Broad-Spectrum Inhibition of Retroviral and Filoviral Particle Release by Tetherin | 331 | 22.07 | JOURNAL OF VIROLOGY | Jouvenet, Nolwenn | 2009 |
| 10 | Proposal for a revised taxonomy of the family Filoviridae: classification, names of taxa and viruses, and virus abbreviations | 307 | 21.93 | ARCHIVES OF VIROLOGY | Kuhn, Jens H | 2010 |
| 11 | Ecological dynamics of emerging bat virus spillover | 295 | 32.78 | PROCEEDINGS OF THE ROYAL SOCIETY B-BIOLOGICAL SCIENCES | Plowright, Raina K | 2015 |
| 12 | T-cell immunoglobulin and mucin domain 1 (TIM-1) is a receptor for Zaire Ebolavirus and Lake Victoria Marburgvirus | 282 | 21.69 | PROCEEDINGS OF THE NATIONAL ACADEMY OF SCIENCES OF THE UNITED STATES OF AMERICA | Kondratowicz, Andrew S | 2011 |
| 13 | DC-SIGN and DC-SIGNR interact with the glycoprotein of Marburg virus and the S protein of severe acute respiratory syndrome coronavirus | 277 | 13.85 | JOURNAL OF VIROLOGY | Marzi, A; Gramberg, T | 2004 |
| 14 | Marburg Virus Infection Detected in a Common African Bat | 259 | 15.24 | PLOS ONE | Towner, Jonathan S | 2007 |
| 15 | Properties of replication-competent vesicular stomatitis virus vectors expressing glycoproteins of filoviruses and arenaviruses | 259 | 12.95 | JOURNAL OF VIROLOGY | Garbutt, M | 2004 |

Ranking: according to the number of total citations; AC, Average citation
